# Prevalence of Chronic Periodontitis and Chronic Stress in the South Indian Population

**DOI:** 10.7759/cureus.33215

**Published:** 2023-01-01

**Authors:** Smrithi V Varma, Sheeja Varghese, Sajan V Nair

**Affiliations:** 1 Department of Periodontology, Saveetha Dental College, Chennai, IND; 2 Department of Orthodontics, Sri Sankara Dental College, Trivandrum, IND

**Keywords:** mental health, epidemiology, oral health, psychoanalytical parameters, chronic stress, chronic periodontitis

## Abstract

Background

Chronic stress is commonly thought to have a net negative effect on the efficacy of the immune response, leading to an imbalance between host and parasite and consequently resulting in a periodontal breakdown.

Aim

To identify the prevalence of chronic periodontitis and chronic stress as well as a comparative evaluation of clinical, demographic, and psychoanalytical parameters among the South Indian population.

Materials and methods

A total of 500 subjects between the ages of 30 and 60 were chosen from the Trivandrum district, Kerala, using multistage random sampling. Subjects were evaluated based on psychoanalytical parameters as well as periodontal examination. Psychoanalytical parameters were measured by the questionnaire method using the perceived stress scale. Periodontal parameters examined were the probing depth, clinical attachment loss, bleeding on probing, simplified oral hygiene index, and community periodontal index (loss of attachment).

Statistical Analysis

Categorical and quantitative variables were expressed as frequency (percentage) and mean ± SD respectively. Logistic regression analysis was used to analyze the association between the periodontal variables and psychoanalytical variables. All the statistical analysis was performed using IBM Statistical Package for Social Sciences (SPSS) Statistics for Windows (IBM Corp., USA).

Results

A total of 500 subjects, of whom 308 (61.6%) were female and 192 (38.1%) were male, participated in this study. The overall prevalence of periodontitis among all the subjects was found to be 42.4%, and the proportion of periodontitis among the stressed participants was found to be 46.2%, which is 10% higher compared to the non-stressed (36.1%) participants.

Conclusion

Our study showed an increased frequency of periodontitis among the stressed subjects as compared to the non-stressed subjects. These findings suggest that there is a positive association between chronic stress and chronic periodontitis, but further prospective studies are required to establish the extent of the effect chronic stress has on chronic periodontitis and vice versa.

## Introduction

Chronic periodontitis, being the most common oral health concern in both developing and developed nations, has been observed as one of the reasons for tooth loss. Globally, the prevalence of chronic periodontitis has been reported to be 5%-15% of the general adult population [1]. Chronic periodontitis is a complex disease of infectious origin characterized by chronic inflammation and destruction of the supporting structures of the teeth, which is attributed to the presence of bacteria in the dental plaque [2]. There are a number of risk factors that can modify the host's response, thereby modifying the disease's progression, such as smoking, diabetes mellitus, cardiovascular diseases, and stress. However, in this study, one of the key domains that we intended to examine is chronic stress, considering that chronic periodontitis and chronic stress have a bidirectional relationship [3].

Chronic stress is generally thought to have a detrimental effect on the responsiveness of the immune system, which can result in a disparity between the host and parasites, bringing about periodontal breakdown [4]. Studies have shown that psychological stress or other psychosomatic conditions can trigger immune responses that facilitate or aggravate changes in the oral cavity, such as periodontitis [5, 6].

Though there are established predisposing factors for periodontitis, the complete variability of the effect of chronic stress on periodontitis remains unclear. Scientific evidence for chronic stress as a causative factor for periodontitis is fragmentary. This study was conducted in the southern Indian state of Kerala, near Thiruvanthapuram, and aimed to identify the prevalence of chronic periodontitis and chronic stress in the adult population. The study also explores the clinical, demographic, and psychoanalytical factors relating to chronic periodontitis and chronic stress in the study population.

## Materials and methods

This study was conducted in the southern district of the state of Kerala, Thiruvanthapuram, using a cross-sectional study design. To get a representative sample of urban, semi-urban, and rural areas, three panchayats (local administrative units) representing rural areas, one semi-urban area (on the outskirts of the municipal corporation), and one urban locality of the municipal corporation (at the lowest level of the administrative system) were selected using a multistage random sampling method. Initially, three Community Development Blocks (CDB) that are geographically located in the lowland, midland, and highland of Thiruvanthapuram district and have relatively similar socioeconomic characteristics were identified before the random selection of panchayats from each CDB. After identifying the panchayats, one ward was selected randomly from each panchayat. For the current study, the primary sampling unit was the ward. Each CDB has four to five panchayats, and each panchayat has 13 to 23 wards with a population of 1500 to 4000. In the urban area, an urban and semiurban ward under the municipal corporation was randomly selected from the corporation register. The population in each urban ward ranges from 3000 to 4000. From each ward, 25 houses were selected for the study. The mapping of the house was conducted with the assistance of a health worker working in the study areas. Every fifth house was identified from the panchayat register, and the households were interviewed and examined for their oral health status.

Subjects in the age range of 30 to 60 years who were willing to participate and at least had 12 teeth present during the time of the interview were selected for the study. This study was performed with a sample size of 500 subjects, which was approved by the Institutional Human Ethical Committee (IHEC Ref No: SDC/Ph.D.-01/19/10). Subjects who were on treatment for systemic illness, those on cytotoxic or immunosuppressive therapy, edentulous patients, mentally challenged patients, and pregnant patients were excluded from the study. Interview questionnaires were conducted to obtain information related to demographic, social, and economic characteristics, general and oral health conditions, habits and lifestyles, medical and dental histories, and access to oral health care. Furthermore, each participant completed a "perceived stress scale" (PSS) survey for stress assessment.

Periodontal examination

All clinical measurements were recorded by one examiner, a qualified periodontist, using a CPITN-C probe and a mouth mirror. These included: 1) probing depth; 2) clinical attachment loss (CAL); 3) bleeding on probing; 4) a simplified oral hygiene index; and 5) a community periodontal index (loss of attachment). Measurements were recorded at six sites around each tooth in the oral cavity, excluding the third molar, and rounded to the nearest 0.5 mm. The probing pocket depth was obtained by measuring the gingival margin at the deepest region of the probe's penetration. The clinical attachment level of each site was recorded by adding the probing pocket depth and the gingival recession measurements. Bleeding on probing was established by observing its occurrence up to 10 seconds after the examination of probing depth. Indices recorded include the Simplified Oral Hygiene Index for evaluating oral hygiene and the Community Periodontal Index for the loss of attachment. Four posterior and two anterior teeth were examined for chronic periodontitis and oral hygiene. Each index tooth was given a score based on the community periodontal index (code 0: healthy periodontium; code 1: gingival bleeding on probing; code 2: the presence of supra or subgingival calculus; code 3: a periodontal pocket of 4 to 5 mm; code 4: a periodontal pocket of more than 6 mm). CPI is advocated by the World Health Organization (WHO) as a validated tool that can be used in epidemiological surveys on oral health [7, 8]. In our study, periodontitis was diagnosed based on the 2017 World Workshop on the Classification of Periodontal and Peri-implant Disease and Condition [9], wherein a patient is a periodontitis case if: a) interdental CAL is detectable at >2 non-adjacent teeth, or b) buccal or oral CAL >3 mm with pocketing >3 mm is detectable at >2 teeth.

Psychoanalytical parameters

In the absence of a gold standard for measuring stress, the three approaches to stress assessment are: a) the environmental approach, which assesses the occurrence of demanding events (stressors); b) the psychological approach, which evaluates the individual stressfulness of each stressor; and c) the biological approach, which focuses on the corresponding biological elements of the stress response [10]. Questionnaires and interviews are the main measurement tools of the first two approaches. PSS is the most widely used, such as in studies assessing the stressfulness of events, physical and psychiatric diseases, and stress management programs [11]. The PSS-10 showed adequate reliability and validity, supporting its use in this population, and has been considered the preferred method for evaluating the perception of stress (Appendix A). This instrument has 10 questions with response options ranging from zero to four (0 = never, 1 = almost never, 2 = sometimes, 3 = often, and 4 = always). The questions with a positive connotation (4, 5, 7, and 8) had punctuation as follows: 0 = 4, 1 = 3, 2 = 2, 3 = 1, and 4 = 0. The remaining questions are negative and were added directly. Finally, an arithmetic average was obtained to observe which individuals had stress levels greater than the average scores, thus defining the diagnosis of stress. Subjects who had scores below 25 were categorized as having low or mild stress, whereas those who scored between 26 and 30 were classified as moderately stressed, and those who scored between 31 and 40 were severely stressed.

Statistical analysis

Categorical and quantitative variables were expressed as frequency (percentage) and mean ± SD respectively. Logistic regression analysis was used to analyze the association between the periodontal variables and psychoanalytical variables. For all statistical interpretations, 0.05 was considered the threshold for statistical significance. Statistical analyses were performed using IBM Statistical Package for Social Sciences (SPSS) software, version 20.0.Z.

## Results

The total number of subjects included in the study was 500, and the detailed descriptive statistics are given in Table 1. It shows the distribution of the study population according to background characteristics: females were 308 (61.6%), and males were 192 (38.4%). This study had a higher percentage of females (61.6%) when compared to males (38.4%), of which 99.2% were literate, unlike the 0.8% who were illiterate. The gender distribution of males and females was 192/308 (38.4% and 61.6%, respectively). The mean age of study participants was 44.72 (±9.245). More than half of the study population was employed, whereas 43.2% were unemployed.

**Table 1:** The percentage distribution of the sample according to background characteristics

Background characteristics		Frequency	Percentage	Cumulative Percent
Gender	Male	192	38.4	100.0
	Female	308	61.6	61.6
Education	Illiterate	4	0.8	0.8
	Literate	496	99.2	100.0
Occupation	Absence	284	56.8	56.8
	Presence	216	43.2	100.0
Systemic status	Healthy	302	60.4	60.4
	Diabetic	81	16.2	76.6
	Cardiovascular disease	117	23.4	100.0
Smoking	Absence	445	89.0	89.0
	Presence	55	11.0	100.0
Tooth brushing	Presence	500	100.0	100.0
Periodontitis status	Absence	288	57.6	57.6
	Presence	212	42.4	100.0
Stress status	Absence	298	59.6	59.6
	Presence	202	40.4	100.0

Table 2 represents the percentage distribution of subjects with or without periodontitis against the absence or presence of stress. Out of 202 subjects with stress, 48.5% had periodontitis, whereas 51.5% did not have periodontitis. Out of 212 patients with periodontitis, 46.2% had stress, whereas 53.8% did not. The overall prevalence of periodontitis among all the subjects was found to be 42.4%, and the proportion of periodontitis among the stressed participants was found to be 46.2%, which is 10% higher compared to the non-stressed (36.1%) participants, as shown in Table 2.

**Table 2:** Quantitative analyses of the relationship between periodontitis status and stress status using cross-tabulation

						Stress status					
						Absence		Presence		Total	
Periodontitis status		Absence		Count		184		104		288	
				% within periodontitis status		63.9%		36.1%		100.0%	
				% within the stress status		61.7%		51.5%		57.6%	
		Presence		Count		114		98		212	
				% within periodontitis status		53.8%		46.2%		100.0%	
				% within the stress status		38.3%		48.5%		42.4%	
Total				Count		298		202		500	
				% within periodontitis status		59.6%		40.4%		100.0%	
				% within the stress status		100.0%		100.0%		100.0%	

Table 3 shows the logistic regression analysis of the sample population. Simple descriptive statistics were used to represent the data. A binomial logistic regression was performed to identify the effect of various prognostic factors such as age, gender, smoking habit, stress status, diabetes, and cardiovascular disease status on the odds of developing periodontitis among 500 subjects. The reference categories used in the categorical predictor variables, namely gender, smoking habit, stress status, diabetes, and cardiovascular disease status, were for male and female gender and the absence of the respective conditions. All the statistical analysis was performed using IBM Statistical Package for Social Sciences (SPSS) Statistics for Windows (IBM Corp., USA). A p-value less than 0.05 was considered significant. Of the six prognostic factors included in the logistic regression model, only two were found to be statistically significant (p<0.05): age and smoking status, as shown in Table 3. An increase in age was associated with increased odds of developing periodontitis (OR=1.098, p<0.001), and a smoking habit also increased the odds of developing periodontitis by 2.772 times (p=0.006), which was highly significant. Though there are clinically meaningful differences in the proportion of participants with periodontitis among the two categories of stress status and increased odds of developing periodontitis among the stressed patients (OR = 1.44), the p-value obtained was 0.076 and it falls closer to the alpha margin. Diabetes and cardiovascular disease status did not affect the periodontitis outcome significantly (p>0.05).

**Table 3:** Logistic regression analysis in the sample population B: unstandardized regression weight; SE: standard error; df: degrees of freedom; OR: odds ratio

Independent variables	B	S.E.	Wald	df	p-value	Exp (B) OR	95% confidence interval for Exp(B) Lower Upper	
Gender (Females/Males)	0.351	0.232	2.280	1	0.131	1.420	0.901	2.238
Age	0.094	0.013	54.474	1	< 0.001	1.098	1.071	1.126
Smoking (Absence/Presence)	1.019	0.368	7.663	1	0.006	2.772	1.347	5.704
Stress status (Absence/Presence)	0.368	0.207	3.139	1	0.076	1.444	0.962	2.169
Diabetes (Absence/Presence)	0.140	0.300	0.218	1	0.641	1.150	0.639	2.070
Cardiovascular disease (Absence/Presence)	-0.077	0.126	0.375	1	0.540	0.926	0.724	1.184
Constant	-4.931	0.587	70.514	1	< .001>	0.007		

## Discussion

The results of this cross-sectional study in a representative sample of the South Indian population of 500 subjects indicate an increased prevalence of chronic periodontitis among the stressed participants (46.2%), which is 10% higher than that of non-stressed individuals (36.1%). This is in concurrence with previous reports [5,12, 13]. The biological plausibility for this relationship is based on the understanding that chronic stress results in the activation of the central nervous system, which in turn stimulates the pituitary glands to release the adrenocorticotropic hormone, which activates the adrenal cortex to release cortisol, also known as the stress hormone, which results in decreased immunity, enhancing the likelihood of infections such as periodontal disease.

The prevalence of periodontitis reported in our study was 42.4%, which is consistent with other studies done regarding the same. As per the global burden of disease study that was conducted in 2016, periodontal disease was the 11th most prevalent condition in the world [14]. The high prevalence of periodontal disease observed in this study population was comparable to the observations of other studies done in India and around the world [15,16]. A report by Shah et al. (2007), a multicentric study carried out by the Government of India in collaboration with the World Health Organization to evaluate the prevalence of periodontitis in the Indian population [17], involved a total of 22,400 participants covering both rural and urban districts in seven different states of India. A prevalence of 100% for periodontal disease was reported for the states of Orissa and Rajasthan. In addition, a varied prevalence of attachment loss >3 mm was observed in different states (Maharashtra: 78%, Orissa: 68%, and Delhi: 46%). Studies done worldwide have reported a higher prevalence of periodontitis in developing countries than in developed countries. The periodontitis prevalence observed in a Brazilian rural population ranged between 24.4% and 83%. [18, 19], and in the Thai population, it ranged from 92% to 100% [20]. About 100% of the Vietnamese study population exhibited at least one site with attachment loss [21].

The age group under study was narrowed down to the 30-60 age range, considering that subjects above the age of 30 have gone through several life experiences, including negative life events such as financial problems, overload at work, the death of a partner, personal illness, or retirement. In the present study of 500 subjects, periodontitis was categorized based on age, with 19.4% of the age category between 30 and 41 years, 50.7% between 41 and 50 years, and 63% between 51 and 60 years. The data appears to indicate a higher prevalence of periodontitis in the age group of 51-60 years. These results are in accordance with results reported by Helena et al. in 2019 [22] and Paul et al. in 2016 [23]. This could have been due to various reasons, such as systemic diseases, an increase in the aging population, or the increased retention of natural teeth.

Our study was carried out on a representative sample from an urban and rural district in the state of Kerala. An important finding in this study was the higher prevalence of periodontitis in males (48.4%) as compared to females (38.6%). This finding was similar to the multicentric study done by Shah et al. in 2007 [17], Balaji et al. in 2018 [15], and Abe et al. in 2020 [24]. The higher prevalence of periodontitis seen in men could be due to various attributes such as smoking, alcoholism, and poor oral hygiene. In our study, there was a discrepancy in the number of female and male participants; the numbers of female participants were higher when compared to the male participants; this could have been due to the timing of our study, during which the male participants would have been at work. This could be validated by assigning an equal number of people of the same gender.

Though there is a clinically meaningful difference in the proportion of participants having periodontitis among the two categories of stress status and there were increased odds of developing periodontitis among the stressed patients (OR = 1.44), the p-value obtained was 0.076, which falls close to the alpha margin. Even though stress as an independent variable has a p-value of 0.076, which is not statistically significant, it would be clinically significant.

In our study, smoking showed a statistical significance of (p=0.006) which is comparable to other studies done in this region [25]. With regard to the prevalence of periodontitis in systemic diseases, diabetes is a well-established risk factor for periodontitis. In our study, diabetes increased the odds of developing periodontitis by 1.150 (p=0.641). Although not statistically significant, this is clinically significant, as shown in previous studies [26]. The low statistical significance could have been due to the small sample size.

Limitations

This research, however, is subject to several limitations, such as the fact that this was a cross-sectional study with small sample size. Cross-sectional studies do not establish the temporality of events. Longitudinal studies done with a larger sample size would be necessary to establish a true relationship between stress and periodontitis. It is important to keep in mind the possible bias in these responses due to the higher number of female participants when compared to male participants. The lack of radiographic examination is also considered a limitation of our study. This study is an attempt to reinforce the proposition of this association. In spite of the disparity in the criteria for the classification of periodontal disease and the lack of a universally accepted diagnostic method, epidemiological investigations remain a challenge. This study evaluated all three clinical parameters, namely, bleeding on probing, clinical attachment loss, and probing pocket depth, reducing the possibility of miscategorization of periodontal disease. This pilot study (preliminary study) gives a telescopic understanding of variables and their impact on chronic periodontitis and chronic stress. To the best of our knowledge, no study has been conducted on the prevalence of chronic periodontitis and chronic stress in the South Indian population. Since the confounding factors could be related to both stress and periodontitis, the possibility of an association due to a lack of control cannot be negated. All the potential confounders were adjusted for in this study, thereby reducing their influence on the exposure and outcome. However, this is only a preliminary study with a limited number of participants, the first of its kind in the South Indian population with varied geographical characteristics. Despite the limitations, the relevance of this study cannot be underestimated, as it has touched upon two globally prevalent health conditions. However, to confirm and strengthen the association, more studies are necessary, which can overcome the limitations mentioned in this study.

## Conclusions

The present study showed that among the study population, the overall periodontitis among the stressed subjects was 10% higher than that of the non-stressed subjects. Based on the obtained results, subjects with high levels of chronic stress appeared to be more susceptible to periodontal disease. Stress coping and management strategies, along with periodontal management, could prevent and manage the progression of periodontitis.

## Figures and Tables

**Figure 1:**
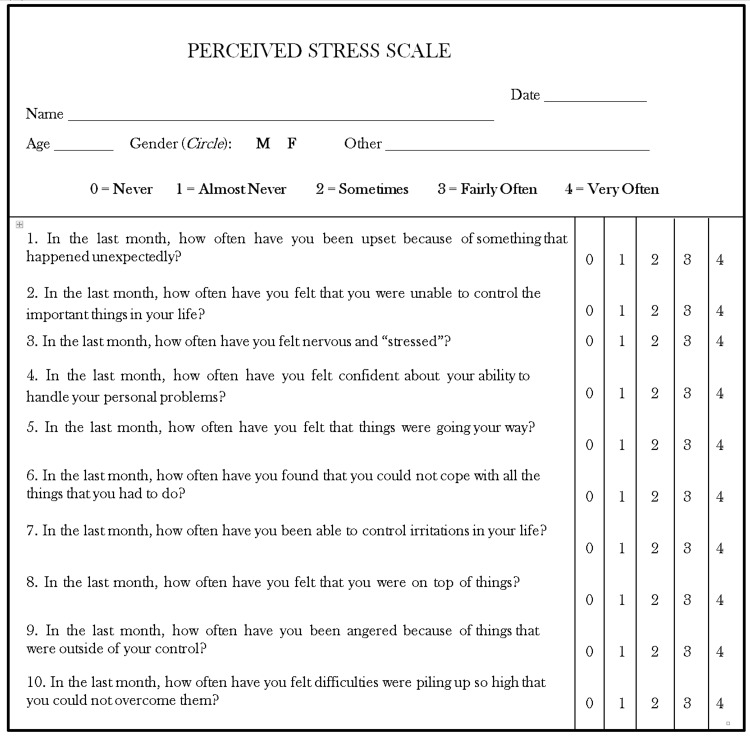
Perceived Stress Scale PSS: Perceived Stress Scale

## Appendices

Appendix A 
